# Target counting with Presburger constraints and its application in sensor networks

**DOI:** 10.1098/rspa.2019.0278

**Published:** 2019-11-06

**Authors:** Sven Linker, Michele Sevegnani

**Affiliations:** 1 Department of Computer Science, University of Liverpool, Liverpool, UK; 2 School of Computing Science, University of Glasgow, Glasgow, UK

**Keywords:** target counting, Presburger arithmetic, sensor networks, model enumeration

## Abstract

One of the applications popularized by the emergence of wireless sensor networks is target counting: the computational task of determining the total number of targets located in an area by aggregating the individual counts of each sensor. The complexity of this task lies in the fact that sensing ranges may overlap, and therefore, targets may be overcounted as, in this setting, they are assumed to be indistinguishable from each other. In the literature, this problem has been proven to be unsolvable, hence the existence of several estimation algorithms. However, the main limitation currently affecting these algorithms is that no *assurance* regarding the precision of a solution can be given. We present a novel algorithm for target counting based on exhaustive enumeration of target distributions using linear Presburger constraints. We improve on current approaches since the estimated counts obtained by our algorithm are by construction guaranteed to be consistent with the counts of each sensor. We further extend our algorithm to allow for weighted topologies and sensing errors for applicability in real-world deployments. We evaluate our approach through an extensive collection of synthetic and real-life configurations.

## 1. Introduction

The recent widespread adoption of wireless sensor network technologies has enabled the development of monitoring and sensing applications deployed over a large number of inexpensive and spatially dispersed devices. The focus of this paper is *target counting*, a sensing task with important applications in domains ranging from farming/agriculture and wildlife protection to traffic and crowd control, indoor security and defence [1,2]. It consists of estimating the total number of observable targets within a region using local counts (also called *readings*) performed by a set of sensors. In this setting, sensors are capable of counting but not identifying targets within their sensing *range*. This implies that multiple sensors may be observing the same target if it is located within the intersection of their overlapping sensing ranges. This may lead to wrong estimates as duplicate observations, together with the inability to distinguish different targets, introduce overcounting. Moreover, it is assumed that the exact position of the sensors and the geometry of their ranges are fully known. This information is referred to as the *topology* of a sensor network.

**Figure 1.**
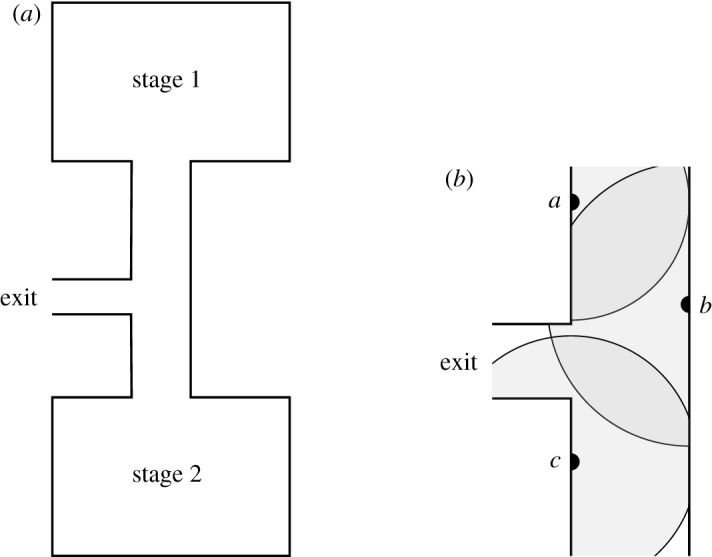
Example application of target counting for crowd control. (*a*) Spatial arrangement of the two stages and a passageway between them leading to the exit and (*b*) detailed view of the sensors deployed in the passageway (*a*, *b*, *c*) and corresponding ranges.

As an example, consider a large event, where crowds sometimes need to be guided through a narrow passageway connecting two stages. Such a situation is depicted in figure 1*a*. For safety reasons (e.g. to prevent overcrowding the passageway and hence outbreaks of crowd crushes), it is necessary to monitor the number of people within this narrow passage. A possible way to carry out this task is to track the mobile phones in the area. However, it is often difficult to use the cellular network information directly to precisely analyse the number of people in the passageway, as each cell is usually too large and covers the entire event venue.

Furthermore, information regarding the participation of individuals to public events might be sensitive (e.g. protests, political rallies). Therefore, the sensors should not send identifiable information about individuals to the base station as it would be the case with mobile phones. Therefore, in these kind of scenarios, we need dedicated sensing devices that do not identify individuals in a given area, but only pass on their aggregate number. This could be achieved, for example, by deploying Bluetooth beacons or passive devices with a restricted range allowing for more precise localization than the cellular network. In this case, the placement of these devices is known, as shown in figure 1*b*.

The core problem we address is that current approaches are not always capable of estimating target counts *accurately* and *reliably*: the range of estimates is often too wide to be usable in practice, and results may even correspond to infeasible *target distributions*.^1^ In the following, we show how this problem occurs in practice by estimating target counts in a simple topology using two current algorithms. A thorough discussion on related work is deferred to §7. Details of the computations performed by the two algorithms considered are reported in appendix A.

**Figure 2.**
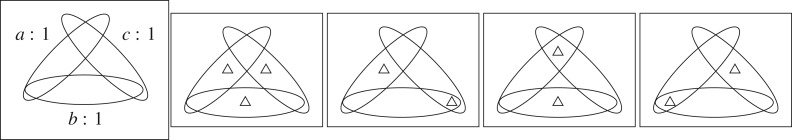
Example topology with three sensors *a*, *b* and *c* and four feasible target distributions.

Consider the example topology with three sensors (*a*, *b* and *c*) in figure 2 (left) where each sensor counts one target within its range (drawn as an oval). Given this configuration, there are four feasible target distributions: one with three targets and three with two targets. These are enumerated in figure 2 (right) where targets are indicated by small triangles. By applying the SCAN algorithm by Gandhi *et al.* [3] to this example, we estimate that the target count is included in the interval [1.5, 3]. Note that the left-hand endpoint of the SCAN estimate falls outside what is feasible, i.e. either two or three targets. Experimental results show that this inaccuracy is further exacerbated in more complex topologies as the estimation interval grows wider with more sensors and more overlapping ranges. By applying the algorithm of Baryshnikov and Ghrist [4] to the same topology, the estimated target count is zero, which does not correspond to any feasible distribution. Experimental results in a study by Pianini *et al.* [5] have also confirmed the difficulty in obtaining reliable results with this algorithm.

In this paper, we propose a novel counting algorithm that overcomes these problems. We do so by computing target counts by means of exhaustive enumeration of the feasible target distributions in a topology. The algorithm is based on the first-order theory of integers with addition, or *Presburger Arithmetic (PA)* [6], which can be solved efficiently by current ‘satisfiability modulo theory’ (SMT) solving tools. To give an intuition about how the additional information from the algorithm can be of use, consider again the topology in figure 2. Our algorithm computes *all* four feasible distributions and then assigns relative frequencies to the possible target counts. In this case, a count of two targets occurs more frequently than a count with three targets as there are three possible distributions with two targets and only one with three targets. This approach allows to adopt any appropriate statistical measure to estimate a target count.^2^ A further benefit of our formalization is to enable more advanced spatial analysis of sensor topologies; for instance, we can compute the likelihood of a target to be in a given spatial region. This kind of information can be extremely valuable in practice as it can be used to adjust sensor placements to obtain more accurate estimates, to optimize scheduled maintenance, to reduce overall energy consumption, etc.

Finally, we extend the basic algorithm to include richer models of the topology and the sensing hardware. First, we augment topologies with *weights* to gain a finer control on how each feasible distribution affects the estimated target count. Second, we introduce a more realistic sensor model by associating an *error* distribution with each sensor reading.

The contributions of this paper are summarized as follows:

— novel formalization of the target counting problem based on exhaustive enumeration of the feasible target distributions by means of Presburger constraints,
— new algorithms supporting both sensing errors and weighted topologies for applications in real-world settings,
— prototype OCaml implementation of our algorithms^3^ based on the Z3 SMT solver,
— evaluation of our approach against a collection of regular (square grids) and randomly generated topologies.

Our article is organized as follows. We begin in §2 by presenting an algorithm to compute all feasible target distributions and explaining how these can effectively be used to estimate target counts. In §3, an extension of the algorithm for weighted topologies is introduced. The algorithm is then further extended with the support for sensing errors in §4. We evaluate our approach in §5 and discuss future challenges and extensions in §6. Related work is presented in §7, and we conclude our work in §8.

## 2. Target counting using Presburger constraints

In this section, we present an algorithm to enumerate all the feasible target distributions within a space covered by a set of sensors. We will then use these distributions to compute the frequencies of the possible target counts and hence infer their probability. This approach builds upon the previous work [7], where such topologies were specified by a formal model based on the first-order logic. However, a crucial difference with the current approach is that previously, sensor readings were not taken into account.

**Figure 3.**
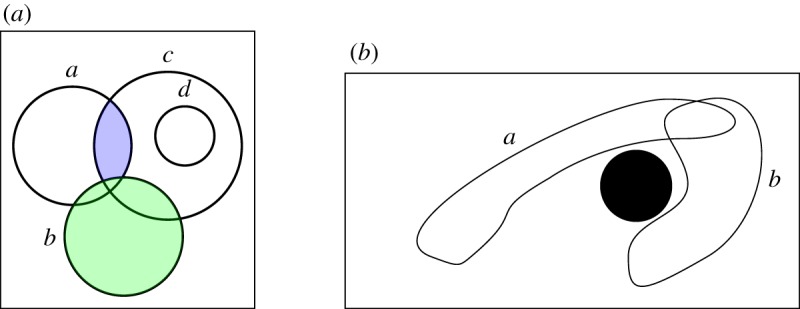
Example topologies. (*a*) Topology with sensors *a*, *b*, *c* and *d*. (*b*) Two sensors (*a* and *b*) having non-convex sensor ranges. The solid black circle represents an obstacle.

We model scenarios following the example sensor deployment shown in figure 3*a*. Sensors are indicated by *a*, *b*, *c*, …, and the corresponding ranges are shown as solid circles. Observe that the sensor ranges partition the space into *zones*, where each zone is defined by the sensor it is covered by. For example, zone {*a*, *c*} denotes the part of the space covered by *both* sensor *a* and *c*, but not by sensor *b*. In this figure, it is coloured in blue. In the formal model, we employ three different sorts, one for the sensors, one for the zones of space and one for the readings of the sensors.

**Definition 2.1 (Topology Model).**

A *topology model* is a structure M=(S,Z,σ,ρ), where *S* is a finite set of sensors, Z⊆P(S) is the set of *zones*, with ∅∉*Z* and, for all *s*∈*S*, there is at least one *z*∈*Z* such that *s*∈*z*. Furthermore, the *range function* σ:S→P(Z) associates a set of zones to each sensor and is defined by *σ*(*s*) = {*z*|*s*∈*z*∧*z*∈*Z*}. The *reading function* ρ:S→N maps each sensor to a reading. For simplicity, we will often omit the braces and commas when we refer to zones. That is, we denote a zone {*a*, *c*} by *ac* or, equivalently, *ca*.

In this section, we assume each sensor yields a single count of targets within its range and, furthermore, that this count is correct. We will modify the definition of *ρ* to widen the scope to non-exact sensor readings in §4.

One of the advantages of this high level of abstraction is that it can be employed in a diverse range of real-life settings. For example, zones can be of any arbitrary shape; in particular, they do not have to be convex, and they can be concave or have holes. This is shown by the example topology in figure 3*b* where the sensor ranges of *a* and *b* are non-convex, since the sensing capabilities are inhibited by the presence of an obstacle, drawn as a solid black circle. By using this notion of spatial models, we can define what we mean by target distributions. Essentially, a target distribution is a function associating each zone with a natural number, which denotes the number of targets residing in this zone.

**Definition 2.2 (Target Distribution).**

Let M=(S,Z,σ,ρ) be a topology model. A map θ:Z→N is a *target distribution* for M. We say that *θ* is *feasible* for M iff $\rho (s)=\sum_{z\in \sigma (s)}\theta (z)$ for all sensors *s*∈*S*. We call *θ* a *partial* target distribution if *θ* is a partial function on *Z*. A partial target distribution is feasible for M if $\rho (s)\geq \sum_{z\in \sigma (s)\cap \mathrm{dom}\theta }\theta (z)$ for all *s*∈*S*. If *θ* is not partial, we also call it *complete*, to emphasize this fact. Let *θ*_1_ and *θ*_2_ be (possibly partial) target distributions for M. We say that *θ*_1_ is an *extension* of *θ*_2_ if dom *θ*_2_⊆dom *θ*_1_ and *θ*_2_(*z*) ≤ *θ*_1_(*z*) for all *z*∈dom *θ*_2_.

Consider again the topology depicted in figure 3*a*. It can be formalized by M=(S,Z,σ,ρ), where *S* = {*a*, *b*, *c*, *d*}, *Z* = {{*a*}, {*b*}, {*c*}, {*a*, *b*}, {*a*, *c*}, {*b*, *c*}, {*c*, *d*}, {*a*, *b*, *c*}}. The sensor range *σ*(*b*) (coloured green) consists of all zones containing *b*, i.e. *σ*(*b*) = {{*b*}, {*a*, *b*}, {*b*, *c*}, {*a*, *b*, *c*}}.

Now consider the following readings for the sensors: *ρ*(*a*) = 1, *ρ*(*b*) = 0, *ρ*(*c*) = 2 and *ρ*(*d*) = 1. Since *b* does not perceive any target, each feasible target distribution *θ* has to satisfy *θ*(*b*) = *θ*(*ab*) = *θ*(*bc*) = *θ*(*abc*) = 0. Furthermore, because *a* reads exactly one target, we also have either *θ*(*a*) = 1 and *θ*(*ac*) = 0, or *θ*(*a*) = 0 and *θ*(*ac*) = 1. In particular, it is not possible that both *θ*(*a*) = 1 and *θ*(*ac*) = 1 because we would then have *ρ*(*a*) = 2. We will exploit this relationship in the next section to compute all feasible target distributions.

### (a) Computing target distributions

**Figure 4.**
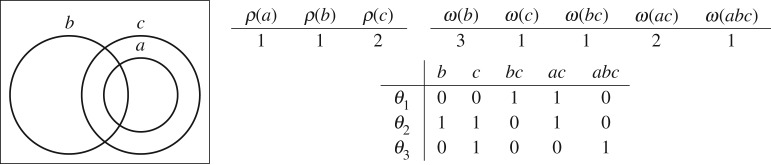
Example topology with sensors *a*, *b* and *c* (left). Reading function *ρ* and weight function *ω* (cf. §3) (top right). Feasible distributions *θ*_1_, *θ*_2_ and *θ*_3_ (right) (bottom right).

The goal of this section is to define formally a procedure for the computation of the set of feasible target distributions for a given topology model M. Namely, we want to find all the ways to place targets in the zones of M while preserving consistency with the readings of M. The most important observation driving our approach follows directly from definition 2.2: the reading of a sensor *s* has to comprise the targets within all the zones in the range of *s*. This becomes apparent in the example in figure 4 where all feasible target distributions (*θ*_1_, *θ*_2_ and *θ*_3_) satisfy this condition. For example, by considering *θ*_1_, we have

$$\begin{array}{rl} & \rho (a)=\theta_{1}(ac)+\theta_{1}(abc)=1+0=1\\ & \rho (b)=\theta_{1}(b)+\theta_{1}(bc)+\theta_{1}(abc)=0+1+0=1\\ \;\mathrm{and}\; & \rho (c)=\theta_{1}(c)+\theta_{1}(bc)+\theta_{1}(ac)+\theta_{1}(abc)\\ & =0+1+1+0=2. \end{array}$$

In the following, we define a constraint satisfaction problem (CSP) over quantifier-free PA formulae to compute all feasible target distributions for a model M=(S,Z,σ,ρ). We assume an infinite set of variables *V* *ar* and associate a variable *x*_*z*_ to each zone *z*∈*Z*. For simplicity, we will omit braces and separators in the names of the variables, i.e. a zone {*a*, *b*} corresponds to *x*_*ab*_. Then, for each sensor *s*∈*S*, we define a constraint as follows:

2.1 $$\rho (s)=\sum_{z\in \sigma (s)}x_{z}.$$

The CSP consists of the set of equations defined above and its solutions are the feasible target distributions for M. Observe that in general, this set is not a singleton, i.e. there may be more than one feasible distribution. For an example, consider figure 4, where the readings of each sensor are given in the table on the right as well as the feasible target distributions. In particular, note that the overall number of targets differs among the distributions: *θ*_1_ and *θ*_3_ have two targets, whereas *θ*_2_ has three.

Solving a single instance of this problem is straightforward: we first create the CSP instance consisting of |*S*| equations as in (2.1) with procedure build - csp, and then we solve them by invoking any off-the-shelf solver supporting PA. However, this does not immediately give us *all* solutions. To that end, the solver needs to be invoked several times, while ensuring that previously found solutions are ignored. Note that any solution *θ* of the CSP corresponds to the formula $\underset{z\in Z}{⋀}x_{z}=\theta (z)$. Hence, to prevent the solver from returning *θ* again at successive invocations, we add the following constraint to the CSP:

$$\mathrm{BUILD}-\mathrm{FORMULA}(\theta )=\underset{z\in Z}{⋁}\neg x_{z}=\theta (z).$$

The complete enumeration algorithm is defined by procedure compute - models in algorithm 1. An analysis of its computational complexity is given in §2c, while a fuller discussion on its scalability in real-world scenarios is presented in §6.


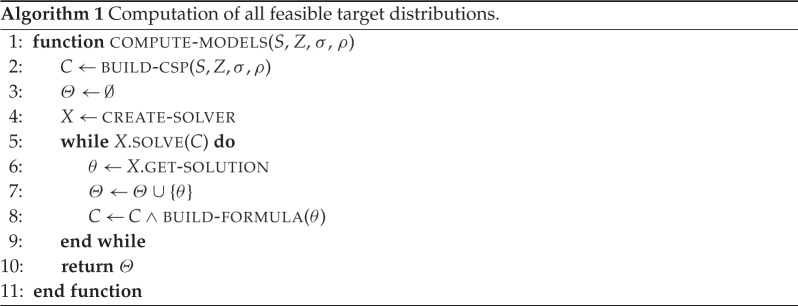


### (b) Frequentist analysis of target distributions

Let $\Theta_{M}$ be the set of feasible target distributions for a model M. For each $\theta \in \Theta_{M}$, we can compute the number of targets present in the space covered by M, simply by summing up the values of the used variables. That is, we can compute the frequency of a target count among all solutions. Hence, we can associate this frequency with the probability of the presence of a certain number of targets within M. For example, from the solutions of the model shown in figure 4, we can deduce that the probability for the presence of exactly two targets is $\frac{2}{3}$, while with probability $\frac{1}{3}$, exactly three targets are present. Furthermore, we can extend this analysis to single zones in the model. This allows for a much more granular spatial analysis. For example, from the solutions of figure 4, we can also deduce that there is a target in zone *ac* with probability $\frac{2}{3}$.

### (c) Complexity

A triply-exponential complexity of the decision procedure for PA was proven by Oppen [8]: in the worst case, the time required to decide the truth of an instance of length *n* is *O*(2^2^2^*n*^^^). Furthermore, Fisher & Rabin [9] gave a non-deterministic doubly exponential time lower bound.

The formulation of our algorithm is based on equations in the form of (2.1) (see procedure build - csp in algorithm 1), which belong to the quantifier-free fragment of PA with a fixed number of variables (one for each zone). This formula class has much lower complexity as it requires only deterministic polynomial time to be decided [10]. A precise bound for equality constraints was defined by Papadimitriou [11] who proved that the complexity is *O*(|*Z*|^|*S*|^(*a*|*S*|)^|*S*|^2^^), a polynomial in $a=\max_{s\in S}\rho (s)$ if, as in our setting, |*S*|, thus |*Z*|, are fixed.

Known results on the complexity of PA concern the decision problem, that is deciding if a set of constraints allows a solution. However, in our setting, all the solutions need to be enumerated, and therefore, we are interested in defining the complexity of the *enumeration problem*. The analysis of algorithm 1 shows that the loop on line 5 is repeated $|\Theta_{M}|=O(2^{a|Z|})$ times, since, in the worst case, the number of solutions to the problem is exponential in the size of the input [12]. At each iteration, a new constraint with |*Z*| variables is generated by procedure build - formula and added to the set of constraints *C*. Therefore, the total running time of algorithm 1 is $O(|Z{|}^{|\Theta_{M}|}2^{a|Z|})$.

## 3. Weighted topologies

The approach described in §2b computes the frequency of a target count, assuming all sensor readings are equally likely. However, many scenarios require us to regard some readings as more important than others. Think for instance of the relative size of sensor ranges in which a larger range is more likely to contain a target as it covers more space than a smaller range. Similarly, sensors in some specific positions in the topology may be known to be more likely to observe targets, for example by analysing historical logs. This is the case, for instance, of an application that counts employees within an office when a sensor covering the area around the door detects targets more frequently, as some employees often enter the office to ask a question, but do not progress beyond the door area.^4^

Consider again the case of the crowd control example presented in §1, i.e. an event where the two main stages are connected by a narrower passageway. In figure 1*b*, we show a possible arrangement of three sensors, *a*, *b* and *c*, in the passageway between the stages. The zones in the stage areas and the passageway can be associated with different weights to reflect the safety focus on the passageway area. This is an established practice in risk analysis of these kinds of scenarios [13]. For instance, the average width of the three different areas can be used to compute the weights of the zones covering them. By assuming stage 1, stage 2 and the passageway have average widths of 17, 23 and 3.5 m, respectively, the weights of the corresponding zones are 3.5÷17 = 0.21, 3.5÷23 = 0.15 and 3.5÷3.5 = 1, that is, every zone in stage 1 has weight 0.21, every zone in stage 2 has weight 0.15 and zones {*a*, *b*}, {*b*}, {*b*, *c*}, {*c*} in the passageway have weight 1.

The weights could also be adjusted over time according to the schedule of the event. For example, during a music event, we can expect a large number of people in the passageway when a popular act finishes on one stage and shortly afterwards another popular band starts on the other stage. Hence, we would increase the weight of the zones {*a*, *b*}, {*b*}, {*b*, *c*} and {*c*} in the passageway. These weights could also depend on the assumed overlap in the audience. This would increase the probability of target distributions where targets are in these zones (cf. §5b) and thus allow a safety operator to promptly identify possible congestions. Operationally, weights can be updated employing suitable statistical methods or machine learning techniques [14,15] as new data are collected.

This use of weights is highly dependent on the application at hand. In this example, it is important that the system errs on the side of caution, i.e. a high density of targets in the passageway is identified as soon as possible to prevent the occurrence of potentially fatal crushes.

To model this kind of scenarios, we extend our approach by associating each zone with a positive real value, denoting the *weight* of the zone. This is a generalization that easily lends itself to various interpretations depending on the application domain. For example, in figure 4, zone *abc* consists of a smaller area than zone *b*. If we assume that the figure reflects the physical topology, we can represent this fact in the model by ensuring that the weight of *abc* is smaller than the weight of *b*.

**Definition 3.1 (Weighted topology model).**

We extend a topology model N=(S,Z,σ,ρ) by a *weight function* $\omega :Z\to R_{\geq 0}$ associating a weight to each zone. We call M=(S,Z,σ,ρ,ω) a *weighted topology model (based on N)*. Observe that we do not require *ω* to be a probability measure, i.e. the sum of all the weights can be different from 1.

**Figure 5.**
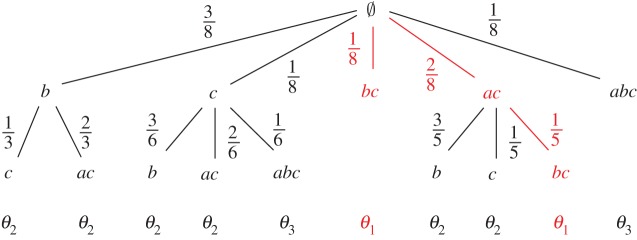
Probability tree for target distributions *θ*_1_, *θ*_2_ and *θ*_3_ for the topology in figure 4. Branches with probability 1 are omitted. The two paths highlighted in red indicate the two possible ways of obtaining distribution *θ*_1_.

The feasible target distributions for a weighted topology model based on M are the same as for M. However, our extension allows us to carry out a more sophisticated analysis of the probability of each distribution by constructing a *probability tree* that represents all the possible ways each distribution can be obtained. For example, consider the topology in figure 4 extended with the weights defined to the right of the figure. Initially, we can place a target in any zone with probability given by the normalized weight of each zone (i.e. the weight of a zone divided by the sum of all the possible weights). Note, however, that our choices to place the next target are limited. For example, if we place a target in zone {*a*, *b*, *c*}, we can only place a target in zone {*c*} afterwards.^5^ On the other hand, if we initially choose {*a*, *c*}, then the next target can be placed in zones {*b*}, {*c*} or {*b*, *c*}. Hence, different choices of placement of a target, thus different probabilities, are possible at each step. The full probability tree for this example topology is given in figure 5.

The probability of a target distribution *θ* can be computed by summing over the probabilities of all the ways to obtain *θ*. These are all the paths in the probability tree from the root to a leaf *θ*. In the example in figure 5, the probability of target distribution *θ*_1_ is given by the sum of the probabilities of the two paths highlighted in red:

$$P(\theta_{1})=\frac{1}{8}+\frac{2}{8}\cdot \frac{1}{5}=\frac{7}{40}=0.175.$$

### (a) Computing the probability tree

Formally, we can create the probability tree as follows. We derive the probability for each target distribution from the possible ways to distribute targets among the zones of the topology model. However, these ways are dependent on each other. For example, in figure 4, if the first target is found in zone {*c*}, then the next target cannot be found in zone {*b*, *c*}. Hence, we need to define a new event space for each random variable dependent on the previous choices. This event space is given by zones *z* of M, for which a target distribution exists, where at least one target is present in *z* and which have not yet been assigned their maximal number of targets. To define the event space, we will use *zone sequences*, which denote the order in which targets have been distributed into the zones. The empty zone sequence is denoted by *ϵ*. Otherwise a zone sequence is $\zeta =\langle z_{1},\ldots ,z_{n}\rangle$. That is, a zone sequence is a function ζ:{1,…,n}→Z. The *size* of *ζ*, denoted by |*ζ*|, is the maximal value of its domain, i.e. |ζ|=max(dom ζ). If *ζ* is a zone sequence and *m* ≤ |*ζ*|, we will denote the sequence coinciding with *ζ* on the domain {1,…,m−1} by *ζ*_<*m*_. We set *ζ*_<1_ = *ϵ*. Furthermore, we denote the *n*th element of *ζ* by *ζ*(*n*). Intuitively, a zone sequence corresponds to a branch of the probability tree, starting at the root and ending at the some node. For example, the branches highlighted in figure 5 are represented by the zone sequences 〈*bc*, *ac*〉 and 〈*ac*, *bc*〉.

We need an auxiliary function to model the addition of a target presence to a target distribution. That is, if the (partial) distribution does not yet contain a value for this zone, we add a presence of one target into this zone, otherwise we increase the current presence by one.^6^

$$\mathrm{inc}(z,\theta )=\left\{ \begin{array}{ll} \theta ⊕[z\mapsto 1] & \mathrm{if}\;z\notin \mathrm{dom}\;\theta \\ \theta ⊕[z\mapsto \theta (z)+1] & \mathrm{otherwise}. \end{array}\right.$$

Each sequence of choices gives rise to an associated (partial) target distribution, which we can compute using recursively the function above.

$$\theta_{\zeta }=\left\{ \begin{array}{ll} \emptyset & \mathrm{if}\;\zeta =\epsilon \\ \mathrm{inc}(\zeta (n),\theta_{\zeta_{<n}}) & \mathrm{if}\;|\zeta |=n. \end{array}\right.$$

Note that different zone sequences may induce the same target distribution. For example, we have *θ*_〈*b*,*c*〉_ = *θ*_〈*c*,*b*〉_. For a sequence *ζ*, we define the still possible target distributions at this point recursively.

$$E_{\epsilon }=\Theta_{M}$$

and

$$E_{\zeta }=\{\theta |\theta \in E_{\zeta_{<|\zeta |}}\wedge \theta \;\text{is an extension of }\;\theta_{\zeta }\}.$$

Note that *E*_*ζ*_ contains only complete target distributions for any *ζ*. Then, for each zone sequence *s*, we identify the set of zones where additional targets may reside, denoted by *Z*_*ζ*_. Intuitively, for each zone *z* in this set, we can still find at least one possible target distribution *θ*, where some targets are in *z*, and either *θ*_*ζ*_ does not already associate a target with *z*, i.e. *z*∉dom *θ*_*ζ*_, or *θ*_*ζ*_ associates less targets to *z* than *θ*. Formally, we have

$$Z_{\zeta }=\{z|\exists \theta \in E_{\zeta }:\theta (z)>0\wedge (z\notin \mathrm{dom}\;\theta_{\zeta }\vee \theta_{\zeta }(z)<\theta (z))\}.$$

Observe that before choosing any zone, the set of possible zones consists of the zones for which there is at least one target distribution, which associates a present target with this zone, i.e. $Z_{\epsilon }=\{z|\exists \theta \in \Theta_{M}:\theta (z)>0\}$. To compute the probabilities for the events at each choice, we need to normalize the weights of the possible zones. To that end, we use *W*_*ζ*_ to denote the sum of the weights of the possible zones after the choices defined by *ζ*.

$$W_{\zeta }=\sum_{z\in Z_{\zeta }}\omega (z).$$

Now, we define random variables *X*_*ζ*_, where *ζ* is the sequence of zones chosen to construct the target distributions. The event space for each *X*_*ζ*_ is $P(Z_{\zeta })$. For each elementary event {*z*}, where *z*∈*Z*_*ζ*_, its probability is given by the weight of *z* normalized by the sum of the weights of possible zones. That is, we set its probability as follows, where *n* = |*ζ*| is the length of *ζ*.

$$P(X_{\zeta }=z|X_{\zeta_{<n}}=\zeta (n-1),\ldots ,X_{\epsilon }=\zeta (1))=\frac{\omega (z)}{W_{\zeta }}.$$

Then, the probability of a specific zone sequence is as follows:

$$\begin{array}{rl} P(\zeta ) & =P(X_{\epsilon }=\zeta (1))\cdot P(X_{\zeta_{<2}}=\zeta (2)|X_{\epsilon }=\zeta (1))\cdot \ldots \\ & =\frac{\omega (\zeta (1))}{W_{\epsilon }}\cdot \frac{\omega (\zeta (2))}{W_{\zeta_{<2}}}\cdot \cdots \cdot \frac{\omega (\zeta (|\zeta |))}{W_{\zeta }}. \end{array}$$

These definitions yield a probability tree similar to figure 5, except that fixed choices (i.e. where only one possibility exists) are omitted in the figure. Then, the probability of a target distribution *θ* is the sum of the probabilities for zone sequences *ζ* with *θ*_*ζ*_ = *θ*.

$$P(\theta )=\sum_{\left\{\zeta |\theta_{\zeta }=\theta \right\}}P(\zeta ).$$

We present a recursive algorithm to create such a probability tree in algorithm 2. The functions extensions(*Θ*, *θ*) compute all extensions of *θ* that are members of *Θ*. Similarly, the function compute - zones(*Θ*, *θ*) returns the zones that are responsible for the existence of these extensions. Both functions can be straightforwardly implemented given the definitions above. The whole function then returns a tree structure, where each node is of the form (*p*, *Θ*, *B*). Such an element denotes that a zone was chosen with probability *p*, the only possible distributions left are in *Θ*, and the next choices are given by the nodes in *B*. A node is a leaf, if it is of the form (*p*, {*θ*}, ∅), where *p* is the probability of choosing *θ* in the final step. We initially call this function with the parameters $\mathrm{tree}(\Theta_{M},1,\emptyset )$.


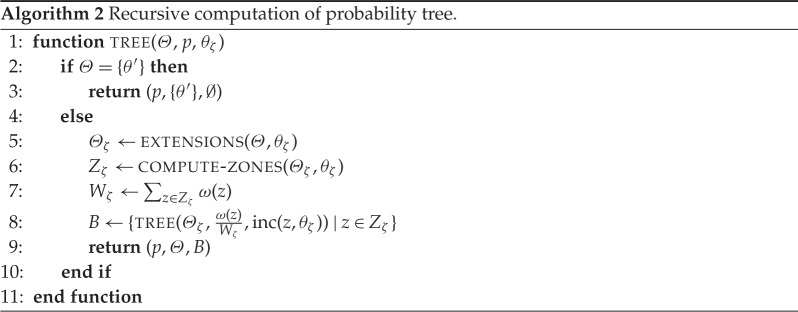


Given a probability tree computed with algorithm 2, we can compute the probability of a zone sequence by following the tree down to the corresponding leaf, and hence, we can also compute the probability of a target distribution in $\Theta_{M}$ by the sum of the corresponding zone sequences.

With this information at hand, we can refine the frequentist analysis of target counts of §2b. Instead of associating the same probability with each target distribution, we can sum the probabilities given by the above construction to take the weights into account. Even further, we can analyse the probability for a specific zone *z* to contain one or more targets by summing the probabilities of all zone sequences containing *z*.

To obtain the probability of a target distribution *θ*, we employ the following recursive function:

$$P_{\theta }((p,\Theta ,B))=\left\{ \begin{array}{ll} p & ,\mathrm{if}B=\emptyset \\ p\cdot \sum_{\begin{array}{c} (p^{\prime },\Theta^{\prime },B^{\prime })\in B\\ \wedge \theta \in \Theta^{\prime } \end{array}}P_{\theta }((p^{\prime },\Theta^{\prime },B^{\prime })) & \mathrm{otherwise}. \end{array}\right.$$

That is, the probability of a distribution *θ* at a node (*p*, *Θ*, *B*) is either *p*, if there are no more possible choices for zones, or it is *p* multiplied with the sum of the probabilities for choosing *θ* in the children of the current node. Hence, if we apply the function *P*_*θ*_ to the root of the tree, it traverses the branches and computes the probability of *θ*.

### (b) Complexity

In every call of tree, we require extensions(*Θ*, *θ*) to check whether every member of *Θ* is an extension of *θ*, i.e. in the worst case, the check has to be performed $|\Theta_{M}|$ times. Within each of these checks, every zone of *θ* has to be compared with the member of *Θ* to be checked. Hence, for each such call, the complexity is $O(|\Theta_{M}|\cdot |Z|)$. The function compute - zones(*Θ*, *θ*) does basically the same comparisons; hence, the complexity of each call is also $O(|\Theta_{M}|\cdot |Z|)$. At each stage of the computation, the tree branches with |*Z*_*ζ*_| successors up to a maximum depth of $b=\sum_{s\in S}\rho (s)$. Hence, we need to create at most |*Z*|^*b*^ nodes of the tree. All in all, the complexity of the whole computation is $O(|Z{|}^{b}\cdot |\Theta_{M}|\cdot |Z|)$. The worst case for computing the probability of a target distribution is that the distribution is at every leaf of the tree. Hence, the complexity of this computation is also *O*(|*Z*|^*b*^).

## 4. Target counting with error bounds

In this section, we extend the enumeration algorithm introduced in §2 to take sensing errors into account by assuming each sensor produces a maximum and a minimum reading instead of a single value. We present the formal definitions for non-weighted topologies in the following section. Subsequently, we introduce weights to these topologies, similar to the extensions shown in §3.

### (a) Topologies with error bounds

To model sensors with error bounds, we define the codomain of the reading function as N×N.

**Definition 4.1 (topology with error bounds).**

We call a structure M=(S,Z,σ,ρ), where *S*, *Z* and *σ* are given as in definition 2.1, and the reading function is ρ:S→N×N, a *topology model with error bounds*. For readability, we denote the first (second) element of the reading of sensor *s* by mins (maxs, respectively). That is, ρ(s)=(mins,maxs).

To acknowledge the inaccuracy in the sensor readings, a feasible target distribution now has to satisfy $\sum_{z\in \sigma (s)}\theta (z)\in [\min s,\max s]$ for every sensor *s*. Hence, we create two constraints for each *s*, instead of one.

4.1 $$\begin{array}{rl} \min s & \leq \sum_{z\in \sigma (s)}x_{z}. \end{array}$$

4.2 $$\begin{array}{rl} \max s & \geq \sum_{z\in \sigma (s)}x_{z}. \end{array}$$

Observe that the constraints for exact sensor readings defined by equation (2.1) are a special case of this definition, i.e. when mins=maxs. With this change, the solutions of the corresponding CSP are again all feasible target distributions for the sensor readings. Note that these solutions have exactly the same form as the solutions for the case with exact sensor readings. In particular, this allows us to extract the frequency of target counts within the solution space in the same way as for exact sensors readings, both for the whole of the space as well as for single zones.

Furthermore, this correspondence allows us to relate feasible target distributions for topologies with error bounds with sensor readings without error bounds. For a feasible distribution *θ*, we can unambiguously compute what the reading for each sensor *s* *should* be. We call this value the *admissible* sensor reading of *s* for *θ*. For example, consider again figure 4, and let *ρ*(*a*) = (1, 1), *ρ*(*b*) = (1, 1) and *ρ*(*c*) = (0, 2). Then, all three target distributions shown in the figure are still feasible. Furthermore, the admissible sensor reading of *c* for *θ*_1_ is 2. However, there cannot be a feasible target distribution *θ* such that 0 is admissible for *c* because *a* has to sense at least one target and its range is a subset of the range of *c*.

**Definition 4.2 (admissible and derived sensor readings).**

Let *θ* be a feasible target distribution for the topology with error bounds M=(S,Z,σ,ρ). Then, the *admissible sensor readings* of *s* for *θ* is $\sum_{z\in Z\wedge s\in z}\theta (z)$. The *sensor readings derived from θ* are given by a function ${\bar{\rho }}_{\theta }:S\to N$, defined by $\bar{\rho }(s)=\sum_{z\in Z\wedge s\in z}\theta (z)$. That is, the derived sensor readings map each sensor to its admissible reading for *θ*. Generally, we will denote derived sensor readings by $\bar{\rho }$.

Note that to compute the set of derived sensor readings, we need to examine M as a whole, or, more specifically, all feasible target distributions for M. Consider the example above, where 0 was not an admissible sensor reading for the sensor *c*. This restriction stems from the zone structure of M and not just from the values of its reading function. Furthermore, we have the following uniqueness property.

**Lemma 4.3.**

*Let* M *be a topology with error bounds and* *θ* *a feasible target distribution for* M. *Then, the sensor readings derived from* *θ* *are a unique function*.

**Proof.**

For each sensor *s*, the admissible sensor reading is unique, as it consists of the sum of all values of *θ* for each zone that is part of the range of *s*. ▪

In particular, this lemma implies that the derived sensor readings partition the set of feasible target distributions. We will exploit this relationship in the following section.

### (b) Weighted topologies with error bounds

The approach for weighted topologies described in §3 can be extended to include sensing errors by introducing an estimation of the error distribution for each sensor. Such information can either be given *a priori* or learned from the historical data by means of Bayesian inference [14] or other machine learning techniques [16].

**Definition 4.4 (weighted topology with error bounds).**

Given a weighted topology model N=(S,Z,σ,ρ,ω), we can extend N further by a probability distribution over the interval of possible readings for each sensor. Hence, to each sensor *s*, we associate a probability distribution $\delta_{s}:[\min s,\max s]\to [0,1]$. We call the model $M=(S,Z,\sigma ,\rho ,\omega ,(\delta_{s}{)}_{s\in S})$ a *weighted topology model with error bounds*.

We do not impose any restrictions on the probability distributions: each *δ*_*s*_ is a function, such that for each r∈[mins,maxs], we have *δ*_*s*_(*r*) > 0 and $\sum_{r\in [\min s,\max s]}\delta_{s}(r)=1$. This means we do not enforce the distributions to be, for instance, normal or exponential.

Since for a given sensor, not every element in the range [mins,maxs] may be admissible, we have to condition the probability of the sensor readings on the only admissible values that can be derived from the set of solutions for M.

If we treat the derived sensor readings as possible events, the whole event space is defined as follows:

$$R=\{{\bar{\rho }}_{\theta }|\theta \in \Theta_{M}\}.$$

We can then condition the probabilities for the derived sensor readings. The probability for a derived sensor reading is the product of the probabilities of the occurrence of each reading:

$$P(\bar{\rho })=\prod_{s\in S}{\mathrm{Dist}}_{s}(\bar{\rho }(s)).$$

Furthermore, we sum all probabilities of the possible events (i.e. the derived sensor readings) to get the value for the whole event space. Note that this may be less than 1, since not every derived sensor reading may be admissible. Formally, we have

$$P(R)=\sum_{\bar{\rho }\in R}P(\bar{\rho }).$$

Finally, the probability that a specific-derived sensor reading occurs depends on the overall event space, and hence, we normalize the probability accordingly.

$$P(\bar{\rho }|R)=\frac{P(\bar{\rho })}{P(R)}.$$

As explained above, the feasible target distributions form a partition according to their derived sensor readings, i.e. $\Theta_{\bar{\rho }}=\{\theta |\theta \in \Theta_{M}\wedge \bar{\rho }={\bar{\rho }}_{\theta }\}$. Now, instead of simply computing zone sequences and proceeding as in §3, we parameterize each of these definitions with a derived sensor reading

$$E_{\bar{\rho },\epsilon }=\Theta_{\bar{\rho }}$$

and

$$E_{\bar{\rho },\zeta }=\{\theta |\theta \in E_{\bar{\rho },\zeta_{<|\zeta |}}\wedge \theta \;\text{is an extension of }\theta_{\zeta }\}.$$

The definitions of §3 can be straightforwardly amended to take these changes into account. For example, instead of *Z*_*ζ*_, we refer to $Z_{\bar{\rho },\zeta }$, which in turn refers to $E_{\bar{\rho },s}$. In particular, instead of computing the probability of a zone sequence immediately, we first choose a derived sensor reading. Otherwise, we proceed as before:

$$P(\bar{\rho },\zeta )=P(\bar{\rho }|R)\cdot P_{\bar{\rho }}(\zeta ),$$

where $P_{\bar{\rho }}(\zeta )$ is computed as *P*(*ζ*) in §3, but where all occurrences of zone sequences are preceded by the choice of $\bar{\rho }$. The probability for a target distribution is then defined as follows:

$$P(\theta )=\sum_{\left\{(\bar{\rho },\zeta )|\theta_{\zeta }=\theta \right\}}P(\bar{\rho },\zeta ).$$

Furthermore, we can use algorithms 1 and 2 to solve this straightforwardly: for a model $M=(S,Z,\sigma ,\rho ,\omega ,(\delta_{s}{)}_{s\in S})$, the solutions *Θ* can be computed by compute - models(*S*, *Z*, *σ*, *ρ*), where the internal function build - csp(*S*, *Z*, *σ*, *ρ*) creates inequalities as in equations (4.1) and (4.2). From this set of solutions, we can compute all possible derived sensor readings *R* and then partition $\Theta_{M}$ accordingly to $\Theta_{/R}=\{\Theta_{\bar{\rho }}|\bar{\rho }\in R\}$. Then, we can compute the probability tree for each equivalence class by calling $\mathrm{tree}(\Theta_{\bar{\rho }},P(\bar{\rho }|R),\emptyset )$ for each $\Theta_{\bar{\rho }}\in \Theta_{/R}$. This gives us the means to compute the probability for each feasible target distribution for M, as before.

## 5. Evaluation

In this section, we present empirical results of applying our approach to several example topologies.^7^ We start with a short description of the implementation. Then, we show with an example, how the addition of weights affects the probabilities of target counts. Finally, we evaluate the performance of algorithm 1 with a set of random and regular topologies.^8^

### (a) Implementation

We implemented our approach using the OCaml programming language [17]. To solve the CSP, we employed the SMT solver Z3 [18], which can be invoked via the ML bindings of its API. Z3 is very well suited for our purposes, since it contains dedicated tactics for quantifier-free linear integer arithmetic, and we only need to address minor implementation details: Z3 does not support natural numbers, but only positive and negative integers. Hence, we had to add a constraint *x*_*z*_≥0 for each variable. Furthermore, since we add the negation of every found model to the set of assertions, the CSP increases in size in every step. In our experiments, we found that as soon as the number of solutions exceeds 170 000, Z3 aborts with an exception due to a lack of heap memory. To keep our evaluation reasonable, we hence aborted the search for solutions, if we found at least 100 000 of them.

The computation of the probability tree for weighted topologies was implemented directly in OCaml, with only slight deviations. For example, we did not compute the tree if there was only one possible target distribution.

### (b) Frequentist analysis and weighted topologies

**Figure 6.**
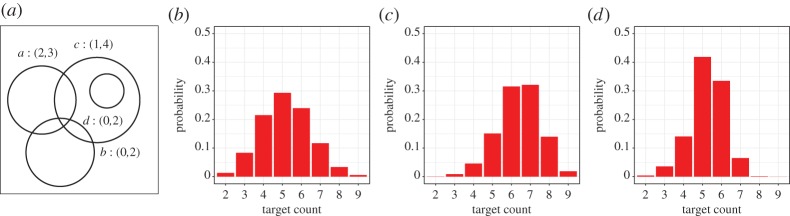
Probabilities of target counts. (*a*) Topology, (*b*) without weight, (*c*) weight: *ω*_1_ and (*d*) weight: *ω*_2_. (Online version in colour.)

In this section, we show how the introduction of weights changes the analysis of target count estimations. We chose an example topology and show how the probabilities of estimated target counts change with the introduction of different weights for zones. The basic topology model of our example is given as in figure 3*a*. However, to increase the number of feasible target distributions, we immediately assume sensor readings with error bounds, as given in figure 6. This topology has 539 feasible target distributions, distributed as shown in table 1. If we use the frequentist analysis as outlined in §2b, we get a probability distribution as shown in figure 6. As the figure shows, the probabilities are uniformly distributed around the target count of 5, which is most probable.

**Table 1.** Target counts for figure 6 (#: number of targets, *Θ*: number of feasible target distributions).

#	2	3	4	5	6	7	8	9
*Θ*	7	45	116	158	129	63	18	3

If we introduce weights into the topology, we also have to fix a distribution over the values of each sensor reading, since we use sensor readings with error bounds. These distributions are given in table 2*a*. We discuss two different weight functions, as shown in table 2*b*. The weights of function *ω*_1_ are all between 0.5 and 3, where the zones only covered by a single sensor, that is, *a*, *b* and *c*, are given weights greater or equal to 2, while the overlaps of the sensor ranges are weighted less (with the exception of *bc*). In particular, this means that distributions with targets in the single zones should be more probable than before. This implies that higher target counts should be more probable, since the amount of overcounting of targets is reduced. Figure 6*c* shows the corresponding probability distribution. As expected, the probability of having six or seven targets is much higher than in the distribution derived only from the number of feasible target distributions.

**Table 2.** Error distributions and weight functions for figure 6.

(a) error distributions for sensors					

	0	1	2	3	4
*δ*_*a*_	—	—	0.2	0.8	—
*δ*_*b*_	0.1	0.5	0.4	—	—
*δ*_*c*_	—	0.3	0.1	0.2	0.4
*δ*_*d*_	0.1	0.1	0.8	—	—

(b) weight functions								

zone	*a*	*b*	*c*	*ab*	*ac*	*bc*	*cd*	*abc*
*ω*_1_	2	3	2	1	1	2	1	0.5
*ω*_2_	0.5	0.1	4	5	5	2	1	5

The weights assigned by function *ω*_2_, however, set a much higher emphasis on the zones denoting the overlaps of the range of sensor *a* with the other sensors. That is, overcounting of targets is more probable, and hence, lower numbers of targets are more likely. This can be seen in figure 6*d*, where the probabilities for the presence of seven or more targets are reduced. However, the probabilities for three and four targets are less than in the frequentist analysis. This is due to the fact that sensor *b* perceiving no targets is less likely than before due to the high weights of zones *ab* and *abc*.

### (c) Random and grid topologies

In this section, we analyse the behaviour of the algorithm to find all feasible target distributions for different sets of topologies. Since the number of distributions tends to be rather high, we do not compute the probability trees for the analysis in this section. Sensor readings may be arbitrarily high, which implies that, generally, there are infinitely many topology models. We restrict our attention to the following topologies.

We used two different types of topology for our experiments. In the following, the *degree of overlap* of a zone *z* is its cardinality. Assume that a set of sensors *S* is given. To start the creation of a topology, we iteratively computed a set of zones, starting with the empty zone. Each time, we chose an existing zone, and, if the degree of overlap of the zone was less than 4, we added one randomly chosen sensor to it. After creating a large set of topologies in this way, we removed all *non-connected* topologies, i.e. topologies where the underlying bi-partite graph is not connected.^9^ From this set of connected topologies with maximal degree of overlap of 4, we chose 160 topologies at random. We repeated this method for each value 5 ≤ |*S*| ≤ 10.

**Figure 7.**
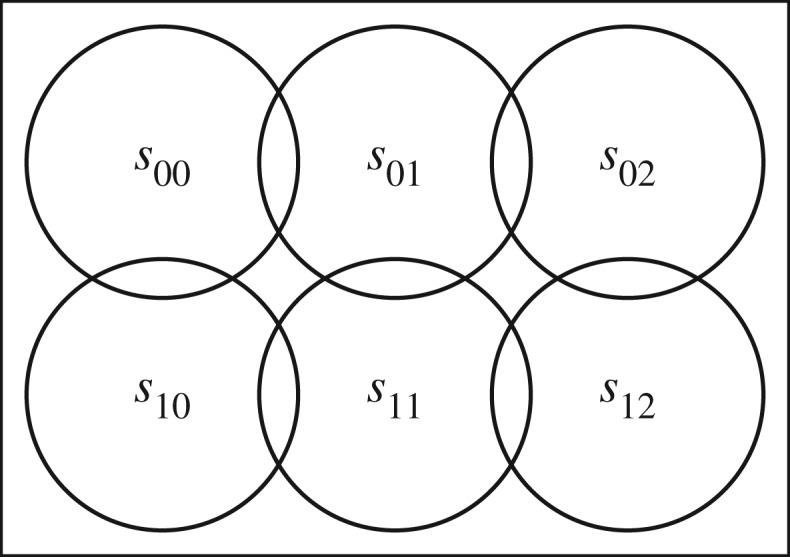
Square topology with two rows, three columns and maximum overlap degree of two.

For a more specialized analysis, we also analysed a *square grid* topology fixing the number of sensors to be 6 and the zones according to figure 7. That is, we take the sensors to be arranged in two rows of three sensors each, and each sensor is overlapping with its neighbours, and no other sensor. This type of topology is similar to some examples analysed by Pianini *et al.* [5].

For both of these types of general topology models, we created readings as follows. We distributed a number of targets uniformly over the set of zones. Then, we derived the reading of each sensor from this given distribution and widen this reading according to a normal distribution. Hence, we always have consistent sensor readings. For each random topology, we created one set of readings from 5 and one from 10 targets in this way. For the square grid topology, we created 80 readings from 5 and 80 readings from 10 targets.

**Figure 8.**
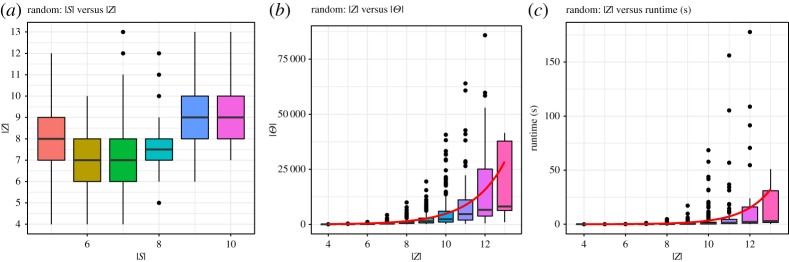
Results for random topologies. (*a*) Degrees of overlap in test set, (*b*) distributions and (*c*) runtimes. (Online version in colour.)

Figure 8*a* shows the interquartile ranges of the size of the sets of zones of the random topologies within the dataset with respect to the number of sensors. This figure shows that most created topologies contain between 7 and 10 zones.

Figure 8*b* shows the relation between the number of distributions and the number of zones in the set of randomly created topologies. In particular, it shows an exponential increase in the number of feasible target distributions. This is consistent with our complexity analysis in §2c. Figure 8*c* shows the relationship between the runtime of algorithm 2 and the size of the zone set. Both diagrams show the exponential influence of the zone set on the number of distributions and the overall runtime. To reduce skewing of the graphs, however, we removed four outliers. Three outliers are due to aborted searches (see §5a), and one was a topology with approximately 91 000 solutions, where the runtime was around 300 s. While these outliers occurred for topologies with a high number of zones (11, 12 and 13), the number of sensors was only either 5 or 7.

**Figure 9.**
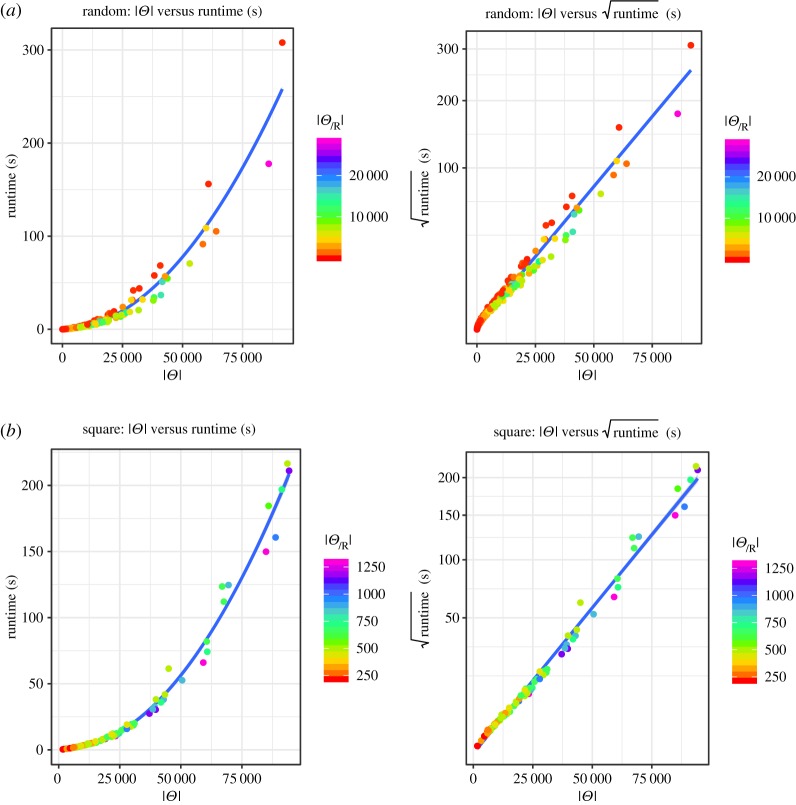
Runtime per number of feasible solutions. (*a*) Random topologies and (*b*) square grid topology. (Online version in colour.)

Figure 9*a* shows how the runtime varies with the number of feasible distributions for our set of random topologies. While the left diagram is a direct comparison, we scaled the *y*-axis in the right figure with the square root of the runtime. This shows that the runtime is polynomial in the number of feasible solutions in our dataset. Similar to the situation above, we removed the outliers from this set of data. Observe that for very low runtimes, the fit is not ideal. We assume that difference is due to the overhead of instantiating the solver and setting up the CSP, in comparison with the very short time the solver needs to run.

For the square grid topology, the corresponding results are shown in figure 9*b*. Here, we removed every data point where the search was aborted, which included almost every run for readings derived from 10 targets. Again, the relationship between the runtime of our algorithm and the number of feasible target distributions appears to be polynomial.

**Figure 10.**
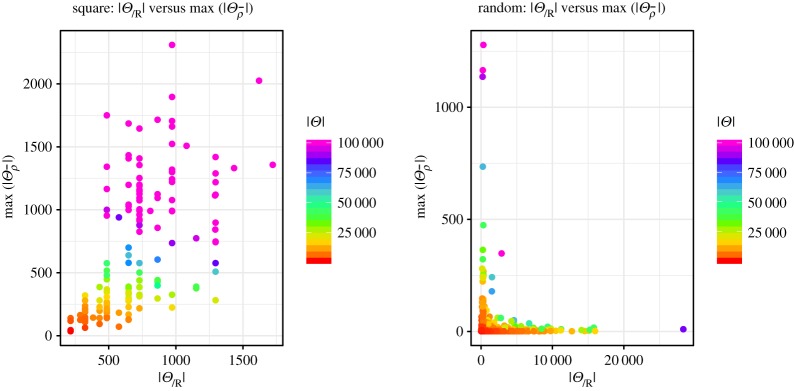
Maximal size of partition block per number of derived readings. (Online version in colour.)

Finally, we show how the partition of the distributions is dependent on the different types of topologies in figure 10. In this figure, we include all data points, since even the aborted searches may show parts of the structure of the space of distributions. From this figure, we can see that the maximal block size of the partition of the feasible solutions for random topologies is typically quite small, even if many different readings are possible. Only for small numbers of derived readings are large numbers of solutions grouped in a single block. This means that the maximal size of the probability tree is more restricted, since only a limited number of solutions may appear on the leaves. For the square topology, however, the maximal block size varies much more, which implies that the computation of the probabilities needs much more space. Furthermore, we can also see that larger number of distributions typically imply more different readings and more feasible distributions that give rise to the same derived reading. Hence, there will be more uncertainty about the correct number of targets in such a topology.

## 6. Discussion

This section discusses future challenges and possible extensions to our approach. The main factors affecting the performance; thus, the applicability of our approach are the number of zones in the topology, the number of feasible target distributions and the size of the probability trees computed by algorithm 2. We discuss them in detail in the following.

### (a) Restrictions due to Presburger arithmetic

The number of zones impacts on the time the SMT solver needs to find each solution, since it determines the number of variables in the CSP. This running time is increased every time a new model for the problem is found, since the CSP to solve is extended by the negated model. Furthermore, the number of feasible target distributions has a direct impact on the applicability of our algorithm. For instance, in our experiments, Z3 ran out of memory with instances with more than 170 000 target distributions. In our setting, such huge number of solutions typically arises for two reasons: a large number of overlapping sensor ranges, where no sensor is completely covered by another (for example square grid topologies, §5), and sensing errors.

Hence, a strategy to increase the applicability of our approach is to aim at reducing the number of feasible target distributions. A straightforward strategy is to ignore the error distributions and only consider some integer close to the mean of each sensor's reading. This would reduce the number of solutions of the CSP, but of course we would omit feasible target distributions, and thus, we would only approximate the probabilities of the target counts. In particular, it is not clear how to quantify the quality of such an approximation.

An alternative way to reduce the number of feasible distributions is to choose only a subset of witnesses (i.e. sampling) for the other computations. However, our application requires these witnesses to be chosen without bias. Chakraborty *et al.* [19] have proposed an efficient algorithm to create almost-uniformly distributed witnesses for Boolean formulae. They employ universal hash functions based on ‘exclusive-or’, and an SMT solver that is optimized for these operators. While this approach is promising for our setting, it is not straightforward to apply it. The main issue is the choice of universal hash functions. The typical families of universal hash functions for integers employ multiplication and modulo operations, and hence, we would leave the language of PA and enter Peano arithmetic, which is undecidable in general. While Z3 still allows us to express these functions, it no longer uses the optimized tactics for Presburger formulae. In an experimental implementation, this approach was too inefficient to be of any use: for a simple topology with nine zones, creating 200 witnesses for feasible target distributions took around 16 minutes. By contrast, the algorithm using only Presburger constraints created all solutions (almost 9500) in around 5 s. Of course, this does not preclude the applicability of such a witness generator, but shows that considerable effort has to be taken. For a different way to generate uniformly distributed witnesses, observe that the formulae defining the set of feasible target distributions define a polyhedron, whose dimension is given by the size of the zone set. Hence, we could employ an algorithm defined by Pak to sample integer points within such polyhedra [20]. However, to the best of our knowledge, no implementation of this algorithm exists yet, so we cannot judge its efficiency in practice.

### (b) Restrictions due to the size of the probability tree

Even with these changes, the probability tree may grow too large to be computed efficiently, since its size is dependent on the maximal readings of sensors and the number of zones. To alleviate this, we may employ standard simulation techniques, simulating the probabilistic choices to distribute the targets among the zones. While this gives us an upper limit on the time necessary for the computation (dependent on the number of simulation runs, i.e. simulated target distributions), it may still be unfeasible when sensing errors are taken into account. In this case, we need separate simulation runs for each possible probability tree, i.e. the number of simulation runs is also dependent on the number of the blocks in the partition *Θ*_/*R*_ as defined in §4b.

### (c) Optimizations

Many of the aforementioned issues can be countered by parallelizing the approach to some degree, with varying success. For example, the computation of probability trees for different equivalence classes of target distributions can be parallelized without any issues, since the trees are fully independent from each other. However, the initial computation of the full set of target distributions is dependent on the capabilities of Z3, and even worse, each step in the computation is dependent on all previous steps, since the CSP changes for each target distribution found. Hence, it is not obvious if and how this step could be parallelized. Furthermore, the size of the probability tree may easily exceed the memory available. In this case, parallelizing the computation does not help either.

Another way to reduce the complexity might be to restrict the possible properties of topologies according to the space they are intended to model. For example, if we required the sensor ranges to be connected, or convex or both, many topologies that we currently accept would not be allowed anymore. Furthermore, we do not distinguish between types of space, for example, whether the surveyed space is two- or three-dimensional Euclidean space. However, it might be possible to enforce properties derived from these types of space onto the topologies, thereby restricting the possible overlaps between sensor ranges and thus the number of zones. However, deciding which types of models are still allowed under these constraints (e.g. if we only want to allow for abstractions of connected subsets of two-dimensional Euclidean space) is generally hard, if it is possible at all (see, e.g. the work of Nenov & Pratt-Hartmann [21], and the surveys in the Handbook of spatial logics [22]).

### (d) Scalability in practice

In the previous sections, we have discussed several general techniques to address the scalability of our approach to larger sensor networks algorithmically. However, in real-world deployments, it is often possible to exploit properties specific to the underlying domain or the actual sensor topology to effectively carry out target counting tasks with our approach even in larger sensor networks.

In applications where maximum coverage is not essential (i.e. when holes in the sensor coverage are tolerable), topologies are typically simpler with only a few signals overlapping with each other.^10^ In this case, the number of zones is linear with the number of sensors, thus rendering the instance of the problem easily solvable. Example applications in this category include environmental sensing scenarios in which sensors are air dropped over a wide landscape [23]. The feasibility of our approach in this kind of scenarios is demonstrated by an experiment involving a synthetic topology formed by 100 sensors and 5 (binary) overlaps in which 14 sensors detect up to one target (thus allowing sensing errors), while the other sensors do not detect any targets. Our algorithm computed all 46 400 target distributions in 38.7 s and partitioned these distributions into 16 384 different sensor readings in 203.1 s. Hence, if the sensor topology is only locally dense, i.e. it exhibits overlaps only over a limited area, while most of the sensor ranges are disjoint, our approach is feasible.

The aforementioned application is also an example for the impact of the number of targets in the system on the performance of our algorithm, i.e. this is another crucial factor affecting its scalability. However, in many applications, low target counts are usually expected, thus allowing the algorithm to scale more easily. For instance, in military applications in which the number of hostile submarines in a region is counted through underwater acoustic sensors, in normal times, individual sensor counts are 0 and rarely exceed 2. We conducted an experiment with the same sparse topology used in the previous experiment but with only 10 sensors detecting one target (allowing sensing errors); our algorithm computed all 2900 solutions in 1.3 s and partitioned them in 3.0 s. As a final example, we chose topologies consisting of 100 sensors, where eight sensors detect targets: two sensors detect between zero and two targets, while the other sensors perceive up to one target. We then increased the number of overlaps between the ranges of these sensors to model a more dense sensor distribution. In our first experiment, we added 21 zones modelling the overlaps of the sensor ranges. Eleven of these zones modelled binary overlaps, while the maximal number of overlapping sensor ranges was six. Our algorithm constructed the 8186 solutions in 6.0 s and also created the partition of 576 blocks of sensor readings in 10.5 s. We then increased the number of zones to 40, where 19 zones were binary, and the maximal number of overlapping sensor ranges was eight. The execution of our algorithm yielded 24833 solutions in 35.7 s, while the partitioning into the 576 blocks took 39.2 s. This shows that even if we increase the density of the topology, our approach can be still feasible if the number of expected targets is low.^11^

Even in large complex topologies, we might have several zones with low weights as, for instance, in the crowd control application described in §3. As a first approximation, it is possible to ‘prune the topology’ by discarding the zones having a weight below a certain threshold and running our algorithm on the reduced topology.

Finally, we note that many applications support or allow target identification. In these cases, our approach should not be used as the problem simply amounts to computing the cardinality of the union of the sets of identifiers produced by each sensor and is thus trivial to solve. Another special case is when the topology has *no overlaps at all*. In this case, employing our approach would be overkill as the target counting problem can be solved by summing over the local counts.

### (e) Extensions

Our approach is open for extensions in several different ways. For example, at the moment, our algorithm only returns target distributions for a single point in time. If we allowed for the passage of time, we can incorporate changes to the topology: if targets move, the readings of the sensors could change, if the sensors move, both the readings and the set of zones could change. In both cases, we could reduce the number of feasible target distributions by requiring that only target counts that were previously feasible stay feasible (under the assumption, that no target enters or leaves the sensed area). The possible ways a topology can change within one time-step is restricted by the possible topological changes [7]. Such an extension could be driven further, by taking weights into account. In particular, it would be possible to use the previously computed feasible target distributions to update the existing weights of the topology. To that end, Bayesian or other learning methods could be employed [14]. This would allow for a dynamically adaptable algorithm.

It remains to be shown how our algorithms can be extended to support different sensor types and thus more applications than target counting. For example, if we had temperature sensors, we would need to associate a value to each zone *z*. A simple idea would be to use the mean of the readings of all sensors covering *z*. However, since temperature is a continuous property of every point in the space, in contrast to the discrete property of the presence of targets, this would be less explicit than the current setting.

Furthermore, we currently assume that all sensors are homogeneous (except for possibly different error bounds) and thus sense the same type of data. It would be interesting to analyse how our approach could be extended to cope with sensors that detect different aspects of the same phenomenon. For example, analysing how fusing sensor readings of different types of sensors is a natural extension.

Currently, we expect our analysis to be carried out on an existing set of deployed sensors. However, this analysis can also be helpful during the design phase of the sensor network to evaluate how different topologies perform in different situations. To that end, we would fix the interesting topologies and define a set of target distributions as typical situations (and possibly some to model extraordinary events). From these distributions, we can derive the sensor readings and then analyse how probable the original distributions were in each sensor topology.

## 7. Related work

The target counting problem has been studied with different assumptions on both the sensor capabilities and the topology of the underlying space [1]. For example, the sensors could return only whether at least *one* target is within their range (binary sensors) [24] or the amount of energy they sense [25,26]. Due to these extensive differences in the approaches, we will only discuss and compare our approach with the most similar algorithms. In particular, we restrict our discussion to sensors that return the *number* of targets within their sensing range. Furthermore, we assume that our sensors have a clearly defined sensing range, that is, we do not take into account that sensing may become less reliable with increasing distance to the sensor.

The approach most similar to ours is the *SCAN* algorithm, as presented by Gandhi *et al.* [3]. The algorithm works in two steps: first, the given topology of sensor ranges is reduced to a *minimal topology*, that is, a topology in which any sensor whose range is entirely covered by other sensor ranges is removed. Second, the sum of the remaining sensor readings and the *maximum overlap degree*, i.e. the maximum number of sensor ranges that overlap in any point in space, are used to derive an estimation interval of the number of targets in the covered space. The SCAN estimate is then defined as the geometric mean of the endpoints of this interval.

We improved on their results in different ways. A precondition of SCAN is the convexity of the sensor ranges, while we allow for arbitrary shapes of the ranges. Furthermore, in two-dimensional space, minimal topologies are not unique [7]. In particular, different minimal topologies may possess different maximum overlap degrees, which in turn impacts the estimation of SCAN. Another peculiar artefact of the SCAN approach is that redundancies in sensor coverage typically *worsen* the estimation, since they can only increase the possible overlap degree. By contrast, our approach is deterministic and uses the redundancies in sensor coverage to its advantage, since the only way a redundancy may impact on our results is by reducing the possible number of target distributions. So, either we get an inconsistent reading, which implies faulty sensors, or our results are more accurate than before.

The bounds computed by our approach are typically tighter than the results of SCAN, and furthermore, they are always realisable, since they are derived from consistent target distributions. By contrast, SCAN may return non-integer results for its bounds (see the example in §1). Furthermore, in addition to bounds on the target counts, we can also more specifically analyse the frequency of the possible target counts, or even their probability, if we allow for weighted topologies. Finally, while the SCAN algorithm can be used to synthesize target distributions in a one-dimensional space, it fails for this purpose in higher dimensions. However, our approach is much more complex due to its reliance on decision procedures for PA, while only simple arithmetic computations are necessary to implement the SCAN algorithm.

Baryshnikov and Ghrist presented a target counting approach based on the analysis of simplicial complexes induced by the topology of the sensors [4]. They proved that their approach is correct and complete under the assumption that a sensor is present at each point *in the continuum*. Hence, applicability of this approach in practice might be limited as already highlighted by the authors. In particular, examples such as the one introduced in §1 are not unlikely to happen in real-world applications. The major downside of this is that generally, the algorithm gives no assurance on the quality of the result. While in the given example, we can immediately see that the computed result has to be wrong (since every sensor detects at least one target), this is just a coincidence. A study by Pianini *et al.* [5] shows huge variation in accuracy depending on topology density and target distribution with more realistic sensor placements. We used topologies similar to their examples to analyse our approach. However, their study also highlights an advantage of the algorithm: it can be implemented in a distributed fashion, on each sensor. Our algorithm, in contrast, can be realistically implemented only on a base-station, since it requires much more computational resources. Furthermore, the formulation of the CSP crucially depends on the availability of *all* sensor readings in one device.

## 8. Conclusion

We have presented a novel approach to solve the problem of target counting. In particular, we examined situations, where sensors may count the number of sensed targets, but cannot identify or distinguish different targets, which leads to overcounting of targets if sensor ranges overlap. In our approach, we abstract from the physical reality of space to a formal model, which allows us to express the problem as formula of PA. The solutions of this formula form all feasible target distributions. This set of solutions can then be analysed with standard statistical methods. Extending the model of space with weights to model the importance and influence of different parts of space allows to extend such analyses further.

The key benefit over existing approaches is that our algorithm guarantees that computed solutions are always feasible. Moreover, it allows for a fine-grained spatial analysis that can be used, for instance, to optimize the topology of a sensor deployment. However, our experiments also show that it is computationally more expensive. In particular, the number of sensors, their positioning in the space to be covered and the size of their sensing ranges have a strong impact on the performance of the algorithm. This limitation can be alleviated by the application of suited sampling techniques to find uniformly distributed witnesses of target distributions. We defer such an extension to future work.

## Figures and Tables

**Figure 11.**
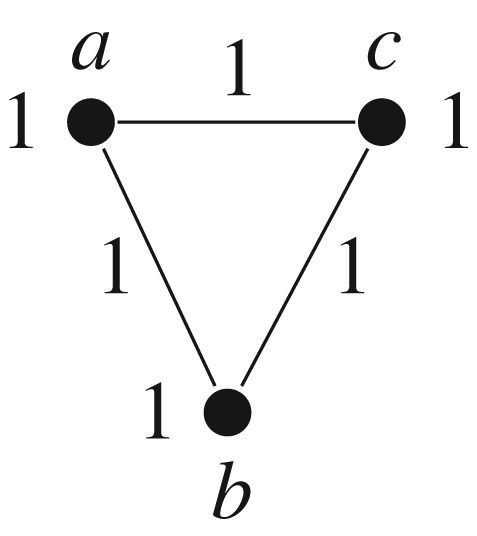
Simplicial complex for topology in figure 2.

## Footnotes

1 A feasible target distribution is a placement of targets in the topology that is consistent with all the individual sensor readings.

2 For instance, the mean target count in this example is 2 × (3/4) + 3 × (1/4) = (9/4) = 2.25 and the median is 2.

3 Source code publicly available at https://bitbucket.org/svenlinker/tcpres

4 This expectation can stem from other data, for example from questionnaires given to the employees or explicit observations.

5 This because *θ*_3_ is the only distribution allowing targets in zones {*a*, *b*, *c*} and {*c*}.

6 We use the notation *f*⊕[*x*↦*y*] to express the *update* of *f* at *x* by *y*. That is, *f*⊕[*x*↦*y*] is the function that assigns *y* to *x* and otherwise coincides with *f* on all elements of dom *f*.

7 The experiments were conducted on a Intel Core i7-7700 CPU running at 3.60 GHz, equipped with 16GB of memory.

8 The implementation, the dataset and the evaluation using the statistical package R can be found at https://bitbucket.org/svenlinker/tcpres.

9 This underlying graph is constructed in the obvious way: The set of nodes is *S*∪*Z*, and there is an edge {*s*, *z*}, if and only if *s*∈*z*.

10 This contrasts, for instance, with regular topologies as the square grid in figure 7.

11 Observe that we did not introduce overlaps for the sensors perceiving zero targets, since they would not increase the complexity of the computation.

## Data accessibility

The implementation, the dataset and the evaluation using the statistical package R can be found at https://bitbucket.org/svenlinker/tcpres. Source code publicly available at https://bitbucket.org/svenlinker/tcpres.

## Appendix A. Target counting with existing approaches

This section describes the steps required to compute estimated target counts for the topology in figure 2 (left) using two current algorithms as described in §1.

**(a) SCAN algorithm**

The given topology is already minimal,^12^ and therefore, no sensor is removed. Then, the maximum overlap degree is computed: *m* = 2. The SCAN estimate is defined by using the individual sensor counts as follows:

$$\hat{t}=\frac{1+1+1}{\sqrt{m}}=\frac{3}{\sqrt{2}}\approx 2.12.$$

Finally, the SCAN estimation interval is given by

$$\left[\frac{\hat{t}}{\sqrt{2}},\hat{t}\sqrt{2}\right]=\left[1.5,3\right].$$

**(b) Baryshnikov and Ghrist algorithm**

The first step consists in creating a simplicial complex starting from the input topology as shown in figure 11. Observe that within this complex, only the nodes and edges (0- and 1-simplices) exist, but the surface within is not part of the complex. The 0-complex is formed by the individual counts of the three sensors: *a* = *b* = *c* = 1. The 1-complex is obtained by drawing an edge between any two sensors with overlapping ranges and then setting the value of each edge as the minimum of its vertices: *ab* = *bc* = *ac* = 1. The Euler characteristic is then computed as an alternating sum for each possible value of *h*, the function associating a value to each cell^13^ of the simplicial complex:

χ{h>0}=#V−#E=3−3=0

and

χ{h>s}=0with s>0.

Finally, the target estimate is obtained by summing the Euler characteristics:

$$\hat{t}=\sum_{s=0}^{\infty }\chi \{h>s\}=0.$$
